# Angio-Seal plug-based versus dual ProGlide for transfemoral hemostasis in transcatheter aortic valve replacement: a systematic review and meta-analysis

**DOI:** 10.1007/s11239-026-03261-z

**Published:** 2026-03-28

**Authors:** Yousef Radwan Alnomani, Mansour Algazar, Mohamed Mosad Omar, Mohamed A. Aldemerdash, Kerollos Henes, Amr Arafa, Bushra Muneer Rageh, Ahmed Abdelaziz

**Affiliations:** 1 Faculty of Medicine, Benha University, Benha, Egypt; 2 Faculty of Medicine, Al-Azhar University, Asyut, Egypt; 3 Faculty of Medicine, Cairo University, Cairo, Egypt; 4 Faculty of Medicine, Sohag university, Sohag, Egypt; 5 Department of Cardiology, El-Mabarra Health Insurance Hospital, Asyut, Egypt; 6 Department of Nephrology, Alexandria University, Alexandria, Egypt; 7 Department of Physiology, Sana’a University, Sana’a, Yemen; 8 Division of Cardiology, Montefiore Health System/Albert Einstein College of Medicine, Bronx, NY USA; 9 Medical Research group of Egypt (MRGE), Cairo, Egypt

**Keywords:** Angio-Seal, Closure device, Perclose ProGlide, Plug-based, Suture-based, TAVR

## Abstract

Transcatheter aortic valve replacement (TAVR) carries risks of vascular and bleeding complications. We aimed to study the effectiveness of Angio-Seal combined with Perclose ProGlide versus dual Perclose ProGlide following transfemoral TAVR. We searched PubMed, Cochrane Library, Scopus, and WOS from inception until November 2024 for studies comparing Parclose ProGlide with Angio-Seal versus dual Parclose ProGlide in transfemoral TAVR. The primary outcome was major vascular complications, while other secondary outcomes were bleeding complications, the need for additional vascular closure device (VCD), minor vascular complications, unplanned surgical intervention, mortality, hematoma, pseudoaneurysm, and dissection. Dichotomous outcomes were pooled and analyzed using odds ratio (OR) with 95% confidence interval (CI) via the DerSimonian-Laird random-effect model. Seven studies (two RCTs and five observational studies) comprising 1,766 patients were included. In RCT-only analyses, single Perclose ProGlide combined with Angio-Seal showed no significant difference compared with dual Perclose ProGlide in major vascular complications (OR = 0.54, 95% CI [0.28–1.04], *P* = 0.07) or major/life-threatening bleeding (OR = 0.66, 95% CI [0.25–1.74], *P* = 0.40). However, it significantly reduced the need for additional vascular closure devices (OR = 0.11, 95% CI [0.05–0.24], *P* < 0.01) and minor vascular complications (OR = 0.52, 95% CI [0.38–0.72], *P* < 0.01). For dual Perclose ProGlide combined with Angio-Seal, no RCTs were available; observational evidence suggested a reduction in major/life-threatening bleeding compared to dual Perclose ProGlide (OR = 0.43, 95% CI [0.21 to0.92], *p* = 0.03), while there was no significant difference between the two groups upon excluding Costa et al. (EuroIntervention, 17:728–735, 2021) (OR = 0.56, 95% CI [0.26 to 1.21], *p* = 0.14). Based on the available RCTs, the combination of single Perclose ProGlide with Angio-Seal during transfemoral TAVR significantly reduces the need for additional vascular closure devices. It may also reduce minor vascular complications, but this effect is not consistently robust. The pooled analysis of observational studies suggests a potential benefit of dual Perclose ProGlide combined with Angio-Seal for major/life-threatening bleeding. However, this finding was not robust in sensitivity analyses and no RCTs have evaluated this strategy.

**Graphical Abstract:**

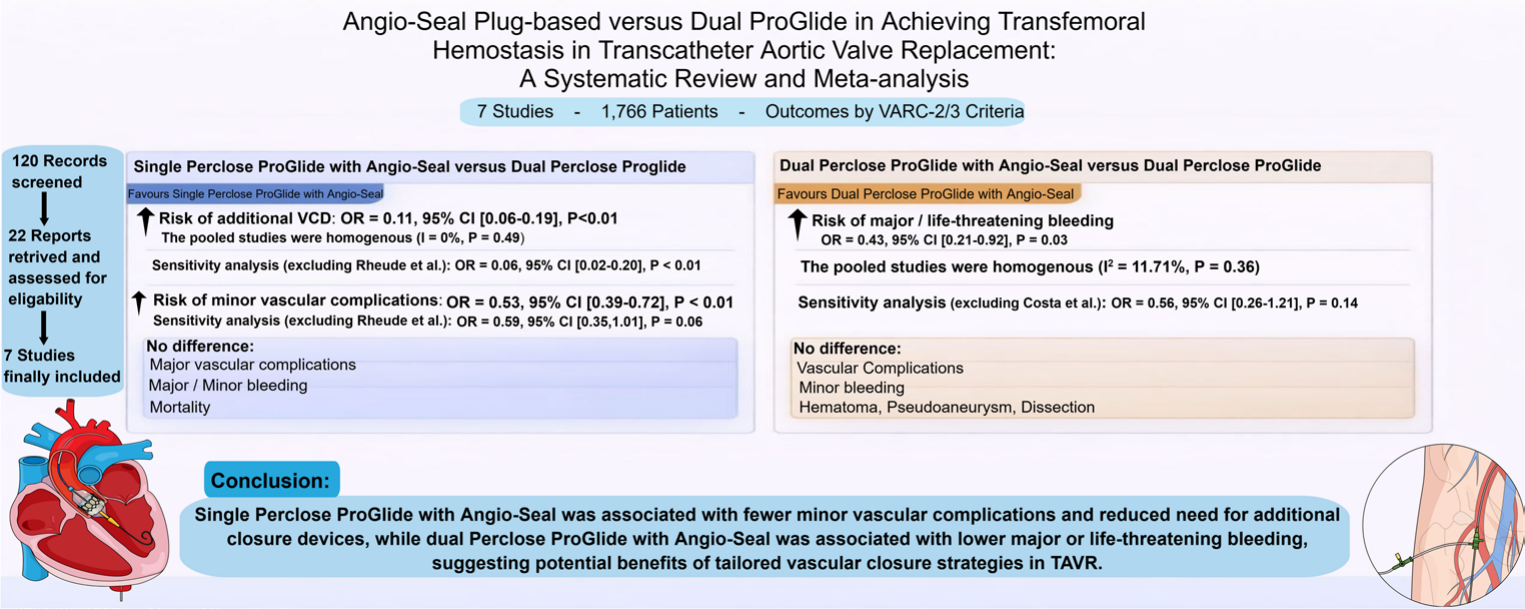

**Supplementary Information:**

The online version contains supplementary material available at 10.1007/s11239-026-03261-z.

## Introduction

Aortic stenosis (AS) is one of the most frequent valve disorders, particularly affecting adults, with nearly 7% of patients aged ≥ 65 years being affected [1]. This condition may be asymptomatic or cause serious symptoms, such as shortness of breath, syncope, angina, and heart failure, which can lead to death [2, 3]. AS may also present with acute decompensation, including episodes of acute heart failure, pulmonary edema, or cardiogenic shock, which are associated with increased short-term morbidity and mortality and often require urgent intervention [4]. Effective management is quite important to alleviate symptoms and improve survival in these patients. Transcatheter aortic valve replacement (TAVR) has become the first choice for elderly patients with severe aortic stenosis conditions, as well as for younger patients with longer life expectancies and lower surgical risks, especially when performed through the femoral route, which is the most common approach due to its minimally invasive technique [5, 6]. Following the procedure of transfemoral TAVR, the femoral access site must be effectively closed to prevent bleeding and vascular complications. Two commonly used devices for this purpose are Parclose ProGlide and Angio-Seal.

Parclose ProGlide is a Suture-mediated closure device (Abbott Vascular, Santa Clara, CA) that has been widely utilized to achieve hemostasis following transfemoral TAVR. It involves placing sutures before dilating the femoral access site, and these sutures are tied immediately after removal of the devices at the end of the procedure [7]. While Angio-Seal is a plug-based device that plays a key role in achieving hemostasis by creating a collagen plug adjacent to the arterial wall, thus creating a mechanical seal [8, 9].

The best approach to achieve long-term hemostasis, especially in large bore arteriotomies, without complications is still unknown [10]. The combination of Parclose ProGlide with Angio-Seal has been considered an approach to achieve hemostasis with fewer vascular complications. However, there are conflicting results between randomized and non-randomized trials comparing Parclose ProGlide with Angio-Seal to dual Parclose ProGlide. Therefore, we aim to rationalize the use of Parclose ProGlide in combination with Angio-Seal versus dual Parclose ProGlide following transfemoral TAVR procedures.

## Methods

We followed the Preferred Reporting Items for Systematic Reviews and Meta-Analysis (PRISMA) statement for preparing this systematic review and meta-analysis [11], and the guidelines proposed in the Cochrane Handbook for Systematic Reviews and Meta-Analysis of interventions (version 5.1.0.). The protocol of this study was prospectively registered on PROSPERO, the international prospective register of systematic reviews (CRD42024617033).

### Eligibility criteria

We included all randomized controlled trials (RCTs) and observational studies that compared the efficacy of Parclose ProGlide with Angio-Seal versus dual Parclose ProGlide following transfemoral TAVR procedures in an intention-to-treat analysis. The studies had to report our outcomes of interest, rates of major and minor vascular complications, bleeding, the use of an additional vascular closure device (VCD), mortality, unplanned surgical intervention, hematoma, pseudoaneurysm, and dissection. The definitions of the outcomes were based on the Valve Academic Research Consortium (VARC-2) or (VARC-3), as shown in (Supplementary Tables 1, 2) [12, 13]. We further excluded non-English studies, conference abstracts, and patients with predominantly high-risk profiles.

### Literature search

A systematic literature search was conducted on the following electronic databases: PubMed, Cochrane Library Central Register of Controlled Trials (CENTRAL), Scopus, and Web of Science (WOS) from inception until November 2024. The search strategy included the following search terms: (“Transcatheter Aortic Valve Replacement” OR “TAVR” OR “TAVI”) AND (“Perclose ProGlide” OR “suture-based” OR “suture-based closure device”) AND (“Angio-Seal” OR “Angioseal” OR “plug-based” OR “plug-based closure device”). The detailed search strategy used in each database and the number of results from each database are detailed in (Supplementary Table 3).

### Screening of the literature search results

All search results obtained from the databases were uploaded to Rayyan for title and abstract screening. Relevant abstracts were retrieved for full-text screening. The studies that met our inclusion criteria were finally included. We also searched the references of the included studies for any additional studies that should be considered for inclusion.

### Data extraction

We extracted data using an online data extraction sheet. The following data were extracted: (1) Characteristics of the study design (year of publication, study location, study design, intervention, comparator, and sample size); (2) Baseline characteristics of the study population (age, sex, body mass index (BMI), hypertension, diabetes mellitus (DM), coronary artery disease (CAD), chronic kidney disease (CKD), peripheral vascular disease (PVD), atrial fibrillation (AF), hyperlipidemia, stroke or transient ischemic attack (TIA), hemoglobin, left ventricular ejection fraction (LVEF), and prior myocardial infarction (MI)); (3) Risk of bias domains; and (4) outcomes (major vascular complications, major bleeding, additional vascular closure device (VCD), minor vascular complications, minor bleeding, unplanned surgical intervention, mortality, hematoma, pseudoaneurysm, and dissection).

### Risk of bias assessment

Two authors (M.M.O and A.A) independently assessed the risk of bias of the included RCTs using the second version of the Cochrane Risk of Bias tool (ROB2), which evaluates the following items: randomization process, deviations from intended interventions, missing outcome data, measurement of the outcome, and selection of reported results. Each study was judged as having a low- or high-risk of bias or some concerns. The quality of observational cohort studies was assessed using the Newcastle Ottawa Quality Assessment Scale (NOS), which evaluates a total of eight items in the following three domains: selectiveness, comparability of cohorts, and outcome assessment. A maximum of one star can be given for each item within the selection and outcome domains, and a maximum of two stars can be given for the comparability domain. Each study was finally judged as being of good, fair, or poor quality. Any disagreements were resolved by a third author.

### Statistical analysis

Dichotomous outcomes were pooled and analyzed as Odds Ratios (OR) with 95% confidence interval (CI) using the DerSimonian-Laird random-effect model in an intention-to-treat analysis. A subgroup analysis was conducted based on characteristics of Parclose ProGlide used (single or dual) in combination with Angio-Seal. We evaluated statistical heterogeneity by visually inspecting the forest plots and measuring their magnitude using the I-square (I^2^) and Chi-square (Q) tests. Significant heterogeneity was considered in case *the p* values for the chi-square test were < 0.1. The I-square test was used to measure the extent of heterogeneity, according to the recommendations of the Cochrane Handbook of Systematic Reviews and Meta-Analysis, with significance defined as I^2^ ≥ 50%. In the case of heterogeneity, a leave-one-out meta-analysis was conducted to ensure that no single study significantly affected the overall effect size.

To test the robustness of the evidence, we conducted sensitivity analysis excluding studies that used another VCD with Angio-Seal, such as ProStyle or Prostar. Subgroup analyses were conducted based on the specific definition criteria (VARC-2 or VARC-3) and study design (RCTs or observational studies). Statistical analyses were conducted using STATA MP version 17 for Windows.

## Results

### Search results

**Fig. 1:**
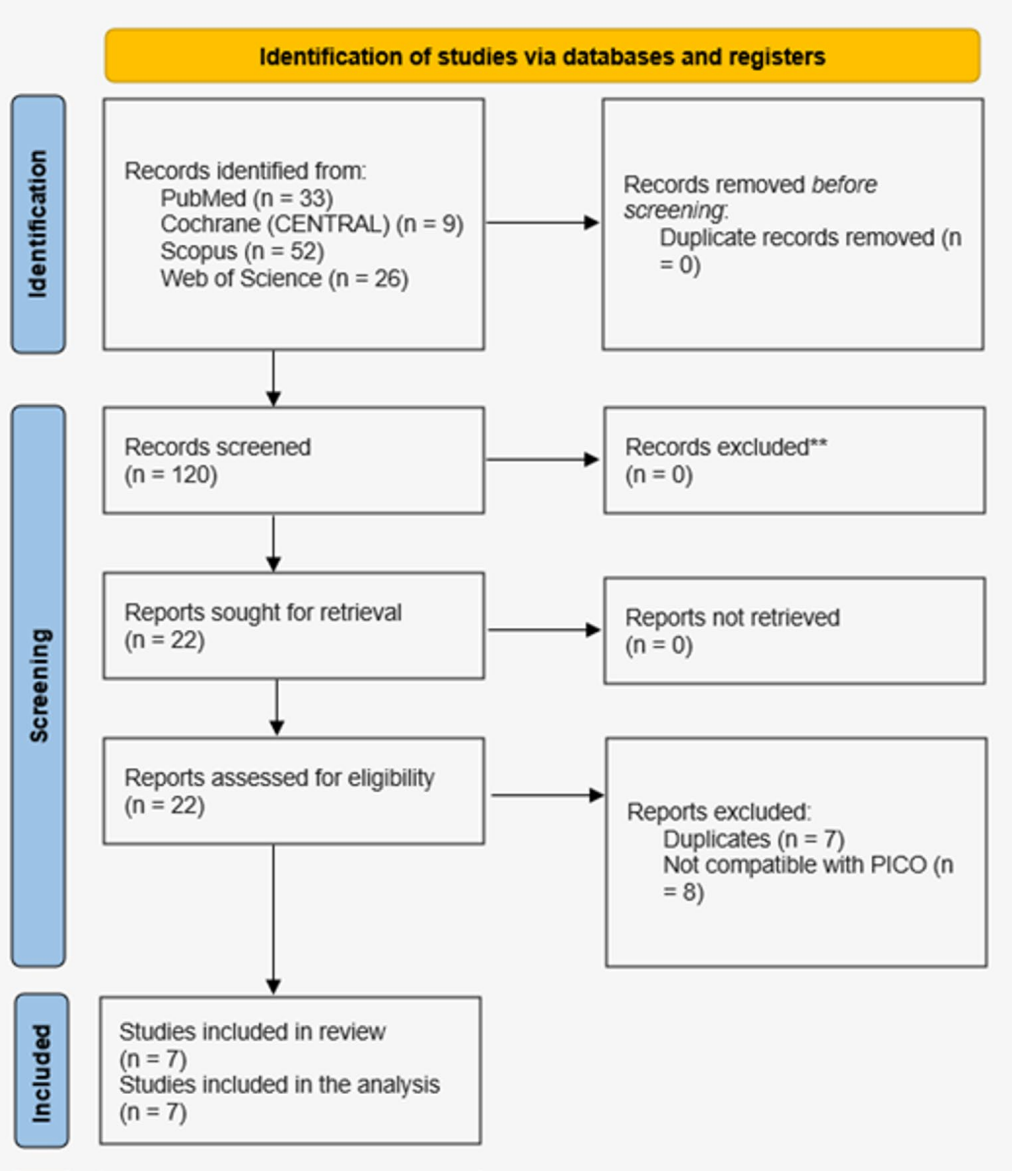
PRISMA flow diagram

A total of 120 articles were retrieved and screened from the databases. After title and abstract screening, 22 articles were eligible for full-text screening. Finally, a total of seven articles (two RCTs and five observational studies) were finally included in the analysis [10, 14–19]. The PRISMA flow chart of the literature search is shown in (Fig. 1).

### Study characteristics

A total of seven studies comprising 1,766 participants were finally included in the analysis. Four studies (two RCTs followed the VARC-3 definition criteria and two observational studies followed the VARC-2 definition criteria), comprising 948 patients, focused on comparing single Parclose ProGlide with Angio-Seal versus dual Parclose ProGlide. Three observational studies, followed the VARC-2 definition criteria, comprising 818 patients, compared dual Parclose ProGlide with Angio-Seal versus dual Parclose ProGlide. The summary characteristics of the included studies and the baseline characteristics of the patients are shown in (Supplementary Table 4).

### Risk of bias assessment

**Fig. 2:**
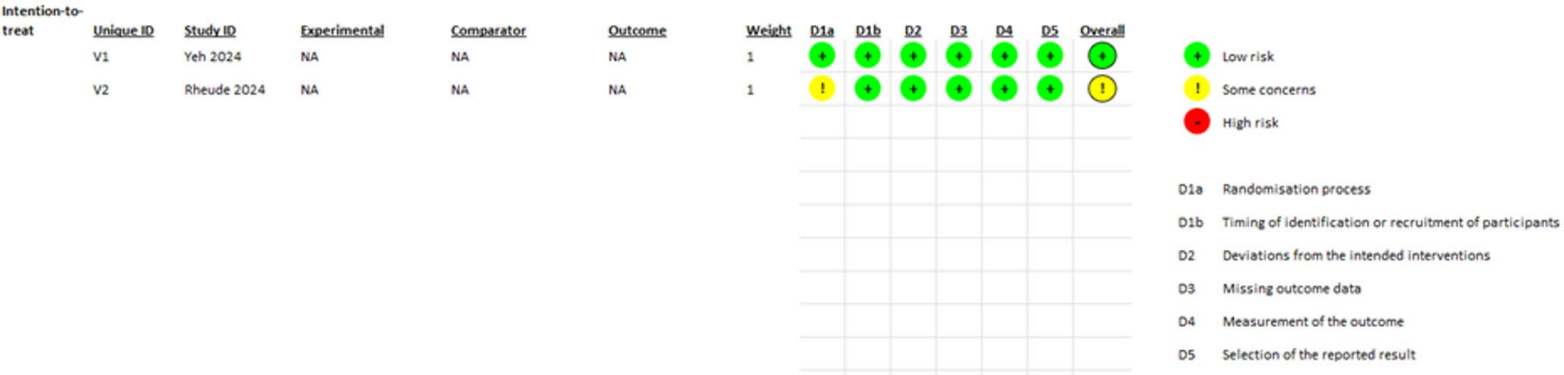
Risk of bias assessment 2 tool (ROB-2)

We used ROB-2 for assessing the two included RCTs. Regarding the study by Yeh et al., it showed an overall low risk of bias, while the study by Rheude et al. showed some concerns regarding the randomization process, which resulted in overall some concerns. The summary of ROB-2 is shown in (Fig. 2).

For the included observational studies, the NOS tool was used, and all studies showed high quality regarding the appropriate selection process, comparability between the two groups, and outcome measurement, as shown in (Supplementary Table 5).

### Primary outcome

#### Major vascular complications

**Fig. 3:**
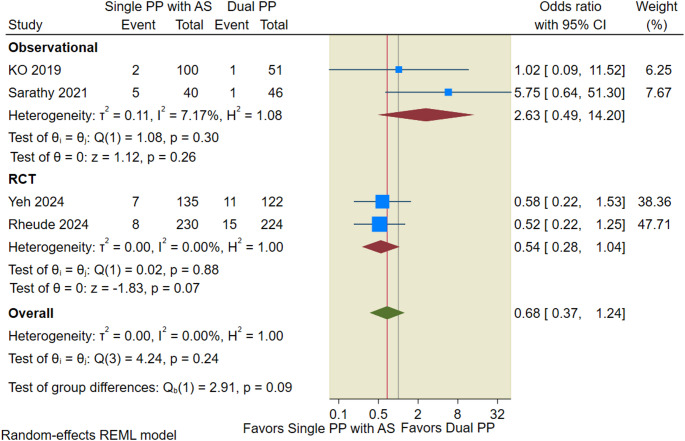
Forest plot of Major vascular complications according to study design

There was no statistically significant difference between single Parclose ProGlide with Angio-Seal and dual Parclose ProGlide in terms of major vascular complications (OR = 0.86, 95% CI [0.37 to 1.24], *p* = 0.21). The pooled studies were homogeneous (I² = 0%, *p* = 0.24), as shown in (Supplementary Fig. 1). Subgroup analysis based on study design showed no significant difference between the two studied groups regarding RCTs (OR = 0.54, 95% CI [0.28 to 1.04], *p* = 0.07), or observational studies (OR = 2.63, 95% CI [0.49 to 14.20], *p* = 0.26), as shown in (Fig. 3). A sensitivity analysis excluding the study by Rheude et al. (which used ProStyle or Parclose ProGlide as suture-based closure devices) did not show a significant difference between the two groups (OR = 1.18, 95% CI [0.3 to 4.68], *p* = 0.82), as shown in (Supplementary Fig. 2).

Dual Parclose ProGlide with Angio-Seal showed no significant difference compared to dual Parclose ProGlide regarding major vascular complications (OR = 0.50, 95% CI [0.20 to 1.26], *p* = 0.14). The studies were homogenous (I² = 41.18%, *p* = 0.19), as shown in (Supplementary Fig. 1). A sensitivity analysis excluding the study by Costa et al. (which used either single Prostar or dual Parclose ProGlide as suture-based closure devices) showed no significant difference between the two groups (OR = 0.76, 95% CI [0.38 to 1.51], *p* = 0.43), as shown in (Supplementary Fig. 2).

### Secondary outcomes

#### Major/life-threatening bleeding

**Fig. 4:**
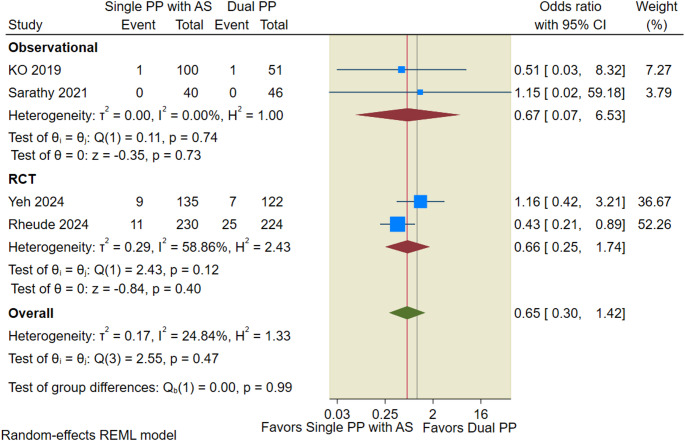
Forest plot of major/life threatening bleeding according to study design

There was no significant difference between Single Parclose ProGlide with Angio-Seal and dual Parclose ProGlide regarding major/life-threatening bleeding (OR = 0.65, 95% CI [0.30 to 1.42, *p* = 0.28). The studies were homogenous (I² = 24.84%, *p* = 0.47), as shown in (Supplementary Fig. 3). Subgroup analysis based on study design showed no significant difference between the two studied groups in either RCTs (OR = 0.66, 95% CI [0.25 to 1.74], *p* = 0.40) or observational studies (OR = 0.67, 95% CI [0.07 to 6.53], *p* = 0.73), as shown in (Fig. 4). A sensitivity analysis excluding the study by Rheude et al. confirmed that there was no significant difference between the two groups (OR = 1.06, 95% CI [0.42 to 2.68], *p* = 0.9), as shown in (Supplementary Fig. 4).

Dual Parclose ProGlide with Angio-Seal was associated with a lower risk of major/life-threatening bleeding than dual Parclose ProGlide (OR = 0.43, 95% CI [0.21 to 0.92], *p* = 0.03). The studies were homogenous (I² = 11.71%, *p* = 0.36), as shown in (Supplementary Fig. 3). A sensitivity analysis excluding the study conducted by Costa et al. showed no significant difference between the two groups (OR = 0.56, 95% CI [0.26 to 1.21], *p* = 0.14), as shown in (Supplementary Fig. 4).

### Additional vascular closure device

**Fig. 5:**
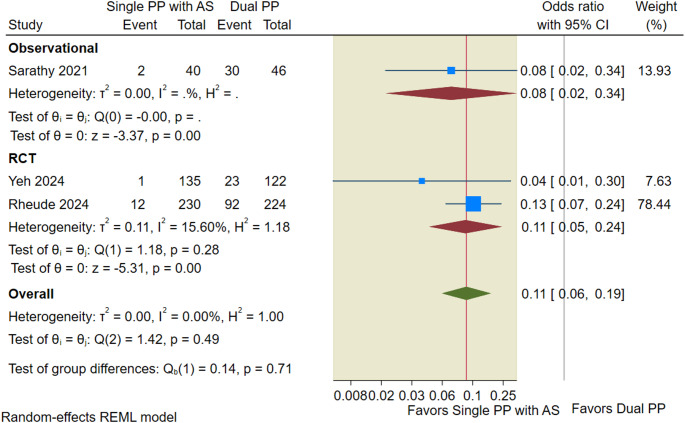
Forest plot of additional VCD according to study design

The pooled OR favored single Parclose ProGlide with Angio-Seal over dual Parclose ProGlide (OR of 0.11, 95% CI [0.06 to 0.19], *p* < 0.01). The studies were homogenous (I² = 0%, *p* = 0.49), as shown in (Supplementary Fig. 5). The absolute event rates were lower in the single Perclose ProGlide with Angio-Seal group (3.7% vs. 36.99%), corresponding to an absolute risk reduction of 33.29%. Subgroup analysis based on study design showed the superiority of a single Parclose ProGlide with Angio-Seal in RCTs (OR = 0.11, 95% CI [0.05, 0.24], p = *p* < 0.01). The absolute event rates were lower in the single Perclose ProGlide with Angio-Seal group (3.56% vs. 33.24%), corresponding to an absolute risk reduction of 29.68%. Also, the single Perclose ProGlide with Angio-Seal group was superior to the dual Parclose ProGlide in observational study (OR = 0.08, 95% CI [0.02, 0.34], p = *p* < 0.01), as shown in (Fig. 5). The absolute event rates were lower in the single Perclose ProGlide with Angio-Seal group (5% vs. 65.2%), corresponding to an absolute risk reduction of 60.22%. A sensitivity analysis excluding the study by Rheude et al. showed a significant reduction in the use of additional VCD in single Parclose ProGlide with Angio-Seal (OR = 0.06, 95% CI [0.02, 0.20], *p* < 0.01), as shown in (Supplementary Fig. 6). The absolute event rates were lower in the single Perclose ProGlide with Angio-Seal group (1.71% vs. 31.55%), corresponding to an absolute risk reduction of 29.84%.

### Minor vascular complications

Single Parclose ProGlide with Angio-Seal was associated with reduced minor vascular complications compared to dual Parclose ProGlide (OR = 0.53, 95% CI [0.39 to 0.72], *p* < 0.01). The studies were homogenous (I² = 0%, *p* = 0.87), as shown in (Supplementary Fig. 7). The absolute event rates were lower in the single Perclose ProGlide with Angio-Seal group (16.24% vs. 32.28%), corresponding to an absolute risk reduction of 16.04%. Subgroup analysis based on study design indicated the superiority of single Parclose ProGlide with Angio-Seal over dual Parclose ProGlide in RCTs (OR = 0.52, 95% CI [0.38, 0.72], p = *p* < 0.01). The absolute event rates were lower in the single Perclose ProGlide with Angio-Seal group (19.45% vs. 37.86%), corresponding to an absolute risk reduction of 18.41%. There was no significant difference between the two groups in observational studies (OR = 0.56, 95% CI [0.23, 1.38], *p* = 0.21), as shown in (Supplementary Fig. 8). The absolute event rates were lower in the single Perclose ProGlide with Angio-Seal group (7.86% vs. 12.37%), corresponding to an absolute risk reduction of 4.51%. After excluding the study by Rheude et al., the pooled OR did not reach significance (OR = 0.59, 95% CI [0.35, 1.01], *p* = 0.06), as shown in (Supplementary Fig. 9). The absolute event rates were lower in the single Perclose ProGlide with Angio-Seal group (10.18% vs. 16.89%), corresponding to an absolute risk reduction of 6.71%.

There was no significant difference between dual Parclose ProGlide with Angio-Seal and dual Parclose ProGlide in terms of minor vascular complications (OR = 0.84, 95% CI [0.37 to 1.90], *p* = 0.67). The studies were heterogeneous (I² = 59.88%, *p* = 0.09), as shown in (Supplementary Fig. 7). The absolute event rates were lower in the dual Perclose ProGlide with Angio-Seal group (10.61% vs. 12.47%), corresponding to an absolute risk reduction of 1.86%. A leave-one-out analysis confirmed that no single study had a disproportionate effect on the pooled OR. Excluding the study by Rheude et al. did not significantly change the results significantly (OR = 1.09, 95% CI [0.46, 2.58], *p* = 0.85), as shown in (Supplementary Fig. 10). The absolute event rates were nearly the same between the two groups (14.57% vs. 14.47%), corresponding to an absolute risk reduction of 0.1%.

### Minor bleeding and unplanned surgical intervention

There was no significant difference between single Parclose ProGlide with Angio-Seal and dual Parclose ProGlide regarding minor bleeding (OR = 1.28, 95% CI [0.89, 1.84], *p* = 0.18), or unplanned surgical intervention (OR = 0.66, 95% CI [0.38, 1.16], *p* = 0.15), as shown in (Supplementary Figs. 11, 12). The studies on minor bleeding and unplanned surgical intervention were homogenous (I² = 0%, *p* = 0.67) and (I² = 0%, *p* = 0.99), respectively. Subgroup analysis based on study design revealed no significant differences between the two studied groups in minor bleeding (RCTs: OR = 1.38, 95% CI [0.93, 2.04], *p* = 0.11; observational studies: OR = 0.77, 95% CI [0.27, 2.15], *p* = 0.61) or unplanned surgical intervention (RCTs: OR = 0.66, 95% CI [0.37, 1.18], *p* = 0.16; observational studies: OR = 0.67, 95% CI [0.07, 6.53], *p* = 0.73), as shown in (Supplementary Figs. 13, 14). A sensitivity analysis excluding Rheude et al. indicated that there was no significant difference between single Parclose ProGlide with Angio-Seal and dual Parclose ProGlide for both minor bleeding (OR = 1.09, 95% CI [0.65, 1.81], *p* = 0.75) and unplanned surgical intervention (OR = 0.66, 95% CI [0.38, 1.15], *p* = 0.14). ), as shown in (Supplementary Figs. 15, 16).

Dual Parclose ProGlide with Angio-Seal did not significantly reduce minor bleeding (OR = 0.95, 95% CI [0.44, 2.05], *p* = 0.90), or unplanned surgical intervention (OR = 0.74, 95% CI [0.29, 1.92], *p* = 0.54) compared to dual Parclose ProGlide, as shown in (Supplementary Figs. 11, 12). The studies on minor bleeding and unplanned surgical intervention were homogenous (I² = 37.74%, *p* = 0.21) and (I² = 0%, *p* = 0.88), respectively. Excluding the study by Costa et al. did not significantly change the results for unplanned surgical intervention (OR = 0.80, 95% CI [0.29, 2.25], *p* = 0.67), as shown in (Supplementary Fig. 16).

### Mortality

There was no significant difference in mortality between single Parclose ProGlide with Angio-Seal and dual Parclose ProGlide (OR = 0.76, 95% CI [0.22 to 2.64], *p* = 0.67). The studies were homogeneous (I² = 0%, *p* = 0.37). Similarly, dual Parclose ProGlide with Angio-Seal did not show significance over dual Parclose ProGlide regarding mortality (OR = 0.71, 95% CI [0.31 to 1.62], *p* = 0.41), as shown in Supplementary Fig. 17. After excluding the study by Costa et al., the pooled OR remained non-significant (OR = 0.79, 95% CI [0.30 to 2.02], *p* = 0.62), as shown in Supplementary Fig. 18.

### Hematoma, pseudoaneurysm, and dissection

The was no significant difference between dual Parclose ProGlide with Angio-Seal and dual Parclose ProGlide regarding hematoma (OR = 1.62, 95% CI [0.42 to 6.27], *p* = 0.48), pseudoaneurysm (OR = 0.48, 95% CI [0.18 to 1.26], *p* = 0.14), and dissection (OR = 1.26, 95% CI [0.63 to 2.53], *p* = 0.52), as shown in Supplementary Fig. 19–21. The studies assessing hematoma were heterogenous (I² = 71.7%, *p* = 0.48), while those assessing pseudoaneurysm and dissection were homogenous (I² = 0%, *p* = 0.88) and (I² = 0%, *p* = 0.37), respectively.

## Discussion

Our meta-analysis aimed to evaluate the efficacy of single and dual Parclose ProGlide with Angio-Seal compared to dual Parclose ProGlide in reducing access-related vascular and bleeding complications after transfemoral TAVR. We pooled data from seven studies, including two RCTs and five observational studies, with a total of 1,766 patients. Our meta-analysis showed that single Parclose ProGlide with Angio-Seal significantly reduced the need for additional VCD and was associated with fewer minor vascular complications compared to dual Parclose ProGlide; however, the difference of minor vascular complications was not consistently observed across sensitivity analyses. On the other hand, there were no significant differences between single Parclose ProGlide with Angio-Seal and dual Parclose ProGlide regarding major vascular complications, major or minor bleeding, unplanned surgical interventions, and mortality. Dual Parclose ProGlide with Angio-Seal was associated with a lower rate of major/life-threatening bleeding compared to dual Parclose ProGlide; however, this finding was not robust in sensitivity analysis and should be interpreted with caution, with no significant differences in major or minor vascular complications, minor bleeding, unplanned surgical interventions, pseudoaneurysm, dissection, and hematoma.

Although two recent clinical trials assessed single Parclose ProGlide with Angio-Seal versus dual Parclose ProGlide and followed the updated outcomes definition of the valvular academic research consortium-3 (VARC-3), which considers more specific and comprehensive criteria than those defined by VARC-2, they reported some inconsistent results [10, 14]. Yeh et al. showed no significant difference between the two groups regarding major and minor vascular complications but favored single Parclose ProGlide with Angio-Seal versus dual Parclose ProGlide, as it was associated with a significant reduction in the need for additional VCD. These findings align with ours, except in minor vascular complications, which may be due to the sheath size used in most patients (16 F and 18 F). In contrast, the study conducted by Rheude et al., which used a smaller sheath size of 14 F in nearly 80% of patients, showed that single Parclose ProGlide with Angio-Seal was associated with reduced major bleeding and minor vascular complications compared to dual Parclose ProGlide. Larger sheaths predispose patients to more vascular complications [20]. A study involving 34,893 patients revealed that sheath sizes larger than 17 F are significantly associated with higher vascular and bleeding complications [21]. Sheath caliber remains one of the most powerful predictors of vascular injury and bleeding complications. Because sheath size was inconsistently reported across the included studies, we were unable to perform stratified or adjusted analyses. Accordingly, residual confounding related to procedural heterogeneity cannot be excluded. Interestingly, the study by Rheude et al. did not depend only on Parclose ProGlide as suture-mediated closure device but also used perclose ProStyle in some patients. ProStyle, a newer variation of Parclose ProGlide, enables the closure of multiple vascular access sites in a single large blood vessel, especially in femoral access site, and is characterized by increased tensile strength needles that makes needle penetration easier through complex anatomy [22, 23].

A study conducted by Costa and colleagues reported a significant reduction in major vascular complications and major/life-threatening bleeding with suture-based devices combined with Angio-Seal compared to suture-based device alone, partially aligning with our findings; however, after exclusion of this study, the association with reduced major/life-threatening bleeding was no longer statistically significant, underscoring the influence of individual studies on pooled estimates. This discrepancy may be due to Prostar was the main suture-based device used in most patients in this study. Although the superiority of Parclose ProGlide compared to Prostyle in TAVR [24, 25], it seems that combining Prostar with Angio-Seal yields better outcomes compared to Prostar alone. We conducted a sensitivity analysis excluding this study, which showed no significant difference in dual Parclose ProGlide with Angio-Seal compared to dual Parclose ProGlide regarding major/life-threatening bleeding.

Antithrombotic therapy may influence vascular and bleeding outcomes following transfemoral TAVR. Most of the included studies reported that unfractionated heparin was routinely administered to maintain adequate intraprocedural anticoagulation, guided by activated clotting time [16]. Protamine was used to reverse heparin before or after vascular closure, with administration either standardized or left to operator discretion. Rheude et al., reported significant differences in protamine administration for heparin antagonization between groups, which may have influenced hemostasis and vascular outcomes [14]. Variations in the strategy of administration of anticoagulation and the reversal may affect bleeding risk, vascular closure success, and the need for additional VCD. However, antithrombotic protocols were not consistently reported across all included studies, limiting the ability to assess their independent impact on vascular outcomes.

A study conducted by Hollowed et al. found that single Parclose ProGlide reduced the postoperative complications, such as arterial dissections and stenosis, more than dual Parclose ProGlide in TAVR [26]. Over the long term, single Parclose ProGlide may be associated with arterial stricture and stenosis due to the high vascular tension it causes, especially in small caliber or calcified femoral arteries [10]. Introducing collagen plug-based Angio-Seal with a single Parclose ProGlide was considered as a potential solution to achieve better outcomes. It is known that factors such as the great experience that operators have in the TAVR procedure, advancements in technology with smaller delivery systems, and minimally invasive techniques such as through percutaneous approach with fewer devices significantly reduce vascular complications. Female gender, sheath size, and peripheral artery disease (PAD) are significant risk factors for vascular and bleeding complications [21]. A recent combination approach using Parclose ProGlide with Femo-Seal was preferred over Parclose ProGlide with Angio-Seal, as it was associated with lower access-related vascular complications, representing a promising strategy for improved hemostasis [27, 28]. Overall, the most consistent finding across analyses was the reduction in the need for additional vascular closure devices, suggesting improved procedural hemostatic success and reduced bail-out interventions. However, convincing evidence for reduction in hard clinical endpoints such as major vascular complications, major bleeding, or mortality remains limited.

### Study Limitations

- Our limitations can be summarized as follows: First, the inability to perform stratified analyses based on sheath size represents a major limitation, as sheath caliber is the strongest determinant of bleeding and vascular complications in transfemoral procedures. Differences in femoral artery anatomy, ultrasound-guided access use, and operator experience may also have influenced outcomes. Second, this is a summary statistics meta-analysis, unlike the individual patient data meta-analysis approach, which reduces bias and enables further standardized analysis. Third, most included studies were observational in nature, which may introduce selection and confounding bias. Fourth, the number of included studies and total sample size remain relatively limited. Fifth, inclusion of both randomized and observational studies within the pooled analysis may introduce design-dependent variability and affect the overall robustness of the conclusions. Sixth, we could not perform a subgroup meta-analysis based on the sheath size used with VCD because of insufficient data. Further trials are warranted to confirm its relationship with the combination closure device approach in achieving hemostasis. Seventh, the small number of included studies limited the ability to assess publication bias using funnel plot analysis or statistical tests such as Egger’s regression test, which require a larger number of studies to provide reliable interpretation. Eighth, secondary vascular access may influence procedural safety and vascular complications. We could not analyze its effect because their limited and inconsistent reporting in the included studies. Ninth, Differences in intraprocedural anticoagulation and protamine reversal strategies may have influenced vascular and bleeding outcomes; however, inconsistent reporting across studies limited further analysis.

### Clinical Implications

- Single Parclose ProGlide with Angio-Seal may be considered when reducing the need for additional vascular closure devices and optimizing procedural hemostasis is a priority, while evidence for reduction in minor vascular complications remains less consistent.
- Dual Parclose ProGlide with Angio-Seal was associated with lower major/life-threatening bleeding rates; however, this finding was not consistent across sensitivity analyses.
- Sheath size (particularly ≤ 14 F) and operator experience are important determinants of vascular and bleeding complication rates.

## Conclusion

Based on available RCTs, the combination of single Perclose ProGlide with Angio-Seal during transfemoral TAVR significantly reduces the need for additional vascular closure devices. It may also reduce minor vascular complications, but this effect is not consistently robust. The pooled analysis of observational studies suggests a potential benefit of dual Perclose ProGlide combined with Angio-Seal for major/life-threatening bleeding. However, this finding was not robust in sensitivity analyses, and no RCTS have evaluated this strategy. Further clinical trials are required to validate our findings.

## Supplementary Information

Below is the link to the electronic supplementary material.

Supplementary Material 1

## Data Availability

All data generated or analyzed during this study are included in this published article.

## Abbreviations

TAVR: Transcatheter aortic valve replacement
VCD: Vascular closure device
OR: Odds ratio
CI: Confidence interval
RCTs: Randomized controlled trials
PRISMA: Preferred Reporting Items for Systematic Reviews and Meta-Analysis
VARC-2: Valve Academic Research Consortium-2
VARC-3: Valve Academic Research Consortium-3
BMI: Body mass index
DM: Diabetes mellitus
CAD: Coronary artery disease
CKD: Chronic kidney disease
PVD: Peripheral vascular disease
AF: Atrial fibrillation
TIA: Transient ischemic attack
LVEF: Left ventricular ejection fraction
MI: Myocardial infarction
CABG: Coronary artery bypass graft
PCI: Percutaneous coronary intervention
ROB-2: Risk of bias tool-2
NOS: Newcastle-Ottawa scale
