# Supplementary material for: Angio-Seal plug-based versus dual ProGlide for transfemoral hemostasis in transcatheter aortic valve replacement: a systematic review and meta-analysis
Source: J Thromb Thrombolysis. 2026 Mar 28;59(5):1167–78. doi: 10.1007/s11239-026-03261-z (PMC13331922; doi:10.1007/s11239-026-03261-z)
Supplement: Supplementary file 1 — Supplementary Material 1 [file 11239_2026_3261_MOESM1_ESM.docx]

# Supplementary Table 1: The Valve Academic Research Consortium-2 consensus document (VARC-2).

| **Major vascular complications** | Access site or access-related vascular injury (dissection, stenosis, perforation, rupture, arterio-venous fistula, pseudoaneurysm, haematoma, irreversible nerve injury, compartment syndrome, percutaneous closure device failure) leading to death, life-threatening or major bleeding, visceral ischaemia, or neurological impairment OR  Distal embolization (non-cerebral) from a vascular source requiring surgery or resulting in amputation or irreversible end-organ damage OR  The use of unplanned endovascular or surgical intervention associated with death, major bleeding, visceral ischaemia or neurological impairment OR  Any new ipsilateral lower extremity ischaemia documented by patient symptoms, physical exam, and/or decreased or absent blood flow on lower extremity angiogram OR  Surgery for access site-related nerve injury OR  Permanent access site-related nerve injury |
| --- | --- |
| **Minor vascular complications** | Access site or access-related vascular injury (dissection, stenosis, perforation, rupture, arterio-venous fistula, pseudoaneuysms, haematomas, percutaneous closure device failure) not leading to death, life-threatening or major bleeding, visceral ischaemia, or neurological impairment OR  Distal embolization treated with embolectomy and/or thrombectomy and not resulting in amputation or irreversible end-organ damage OR  Any unplanned endovascular stenting or unplanned surgical intervention not meeting the criteria for a major vascular complication OR  Vascular repair or the need for vascular repair (via surgery, ultrasound-guided compression, transcatheter embolization, or stent-graft) |
| **Mortality** | \| Cardiovascular mortality   - Any of the following criteria: - Death due to proximate cardiac cause (e.g. myocardial infarction, cardiac tamponade, worsening heart failure) OR - Death caused by non-coronary vascular conditions such as neurological events, pulmonary embolism, ruptured aortic aneurysm, dissecting aneurysm, or other vascular disease OR - All procedure-related deaths, including those related to a complication of the procedure or treatment for a complication of the procedure OR   All valve-related deaths including structural or non-structural valve dysfunction or other valve-related adverse events OR   - Sudden or unwitnessed death OR - Death of unknown cause \| \| --- \| \| Non-cardiovascular mortality  Any death in which the primary cause of death is clearly related to another condition (e.g. trauma, cancer, suicide) \| |
| **Bleeding** | \| Life-threatening or disabling bleeding   - Fatal bleeding *(BARC type 5)* OR - Bleeding in a critical organ, such as intracranial, intraspinal, intraocular, or pericardial necessitating pericardiocentesis, or intramuscular with compartment syndrome *(BARC type 3b and 3c)* OR - Bleeding causing hypovolaemic shock or severe hypotension requiring vasopressors or surgery *(BARC type 3b)* OR - Overt source of bleeding with drop in haemoglobin ≥5 g/dl or whole blood or packed red blood cells (RBCs) transfusion ≥4 units^a^*(BARC type 3b)* \| \| --- \| \| Major bleeding *(BARC type 3a)*   - Overt bleeding either associated with a drop in the haemoglobin level of at least 3.0 g/dl or requiring transfusion of two or three units of whole blood/RBC, or causing hospitalization or permanent injury, or requiring surgery AND - Does not meet criteria of life-threatening or disabling bleeding \| \| Minor bleeding (*BARC type 2 or 3a, depending on the severity)*   - Any bleeding worthy of clinical mention (e.g. access site haematoma) that does not qualify as life-threatening, disabling, or major \|   *BARC: Bleeding Academic Research Consortium; RBC, red blood cell.*  *^a^Given that one unit of packed RBC typically will raise the haemoglobin concentration by 1 g/dl, an estimated decrease in haemoglobin will be calculated.* |

**Supplementary Table 2:** The valve Academic Research Consortium-3 (VARC-3).

| **Major vascular complications** | One of the following:   \| ▪ \| Aortic dissection or aortic rupture \| \| --- \| --- \| \| ▪ \| Vascular (arterial or venous) injury (perforation, rupture, dissection, stenosis, ischaemia, arterial or venous thrombosis including pulmonary embolism, arteriovenous fistula, pseudoaneurysm, haematoma, retroperitoneal haematoma, infection) or compartment syndrome resulting in death, VARC type ≥2 bleeding, limb or visceral ischaemia, or irreversible neurologic impairment \| \| ▪ \| Distal embolization (non-cerebral) from a vascular source resulting in death, amputation, limb or visceral ischaemia, or irreversible end-organ damage \| \| ▪ \| Unplanned endovascular or surgical intervention resulting in death, VARC type ≥2 bleeding, limb or visceral ischaemia, or irreversible neurologic impairment \| \| ▪ \| Closure device failure resulting in death, VARC type ≥2 bleeding, limb or visceral ischaemia, or irreversible neurologic impairment \| |
| --- | --- | --- | --- | --- | --- | --- | --- | --- | --- | --- | --- |
| **Minor vascular complications** | One of the following:   \| ▪ \| Vascular (arterial or venous) injury (perforation, rupture, dissection, stenosis, ischaemia, arterial or venous thrombosis including pulmonary embolism, arteriovenous fistula, pseudoaneurysm, haematoma, retroperitoneal haematoma, infection) *not* resulting in death, VARC type ≥2 bleeding, limb or visceral ischaemia, or irreversible neurologic impairment \| \| --- \| --- \| \| ▪ \| Distal embolization treated with embolectomy and/or thrombectomy, *not* resulting in death, amputation, limb or visceral ischaemia, or irreversible end-organ damage \| \| ▪ \| Any unplanned endovascular or surgical intervention, ultra-sound guided compression, or thrombin injection, *not* resulting in death, VARC type ≥2 bleeding, limb or visceral ischaemia, or irreversible neurologic impairment \| \| ▪ \| Closure device failure[‡](about:blank) *not* resulting in death, VARC type ≥2 bleeding, limb or visceral ischaemia, or irreversible neurologic impairment \| |
| **Mortality** | Cardiovascular mortality  Death meeting one of the following criteria:   \| ▪ \| Related to heart failure, cardiogenic shock, bioprosthetic valve dysfunction, myocardial infarction, stroke, thromboembolism, bleeding, tamponade, vascular complication, arrhythmia or conduction system disturbances, cardiovascular infection (e.g. mediastinitis, endocarditis), or other clear cardiovascular cause \| \| --- \| --- \| \| ▪ \| Intraprocedural death \| \| ▪ \| Sudden death \| \| ▪ \| Death of unknown cause \| \| Valve-related mortality \| \| \| Death presumed to be related to bioprosthetic valve dysfunction[†](about:blank) \| \| \| Non-cardiovascular mortality \| \| \| Death clearly related to a non-cardiovascular cause: such as respiratory failure *not* related to heart failure (e.g. pneumonia), renal failure, liver failure, infection (e.g. urosepsis), cancer, trauma, and suicide \| \| |
| **Bleeding and transfusions** | Overt bleeding[†](about:blank) that fulfils one of the following criteria:  Type 1   \| ▪ \| Overt bleeding that does not require surgical or percutaneous intervention, but does require medical intervention by a health care professional, leading to hospitalization, an increased level of care, or medical evaluation (BARC 2) \| \| --- \| --- \| \| ▪ \| Overt bleeding that requires a transfusion of 1 unit of whole blood/red blood cells[‡](about:blank) (BARC 3a) \|   Type 2   \| ▪ \| Overt bleeding that requires a transfusion of 2–4 units of whole blood/red blood cells[‡](about:blank) (BARC 3a) \| \| --- \| --- \| \| ▪ \| Overt bleeding associated with a haemoglobin drop of >3 g/dL (>1.86 mmol/L) but <5 g/d (<3.1 mmol/L) (BARC 3a) \|   Type 3   \| ▪ \| Overt bleeding in a critical organ, such as intracranial, intraspinal, intraocular, pericardial (associated with haemodynamic compromise/tamponade and necessitating intervention), or intramuscular with compartment syndrome (BARC 3b, BARC 3c) \| \| --- \| --- \| \| ▪ \| Overt bleeding causing hypovolemic shock or severe hypotension (systolic blood pressure <90 mmHg lasting >30 min and not responding to volume resuscitation) or requiring vasopressors or surgery (BARC 3b) \| \| ▪ \| Overt bleeding requiring reoperation, surgical exploration, or re-intervention for the purpose of controlling bleeding (BARC 3b, BARC 4) \| \| ▪ \| Post-thoracotomy chest tube output ≥2 L within a 24-h period (BARC 4) \| \| ▪ \| Overt bleeding requiring a transfusion of ≥5 units of whole blood/red blood cells (BARC 3a) [‡](about:blank) \| \| ▪ \| Overt bleeding associated with a haemoglobin drop ≥5 g/dL (≥3.1 mmol/L) (BARC 3b). \|   Type 4   \| ▪ \| Overt bleeding leading to death. Should be classified as:   \| ▪ \| *Probable:* Clinical suspicion (BARC 5a) \| \| --- \| --- \| \| ▪ \| *Definite:* Confirmed by autopsy or imaging (BARC 5b) \| \| \| --- \| --- \| --- \| --- \| --- \| --- \|   **The timing, indication, and number of transfused blood products should be collected and reported specifically during the index procedure, during the entire index hospitalization, and during follow-up after discharge, whether or not overt bleeding is identified.*  *† Overt bleeding is defined as any clinically obvious source of bleeding or bleeding source identified after appropriate investigation and diagnostic testing (e.g. imaging). Any procedural blood loss should be considered overt bleeding.*  *‡ Total number of transfusions should be reported separately for (i) within 48 h of the index procedure, (ii) the total duration of the index procedure hospitalization, and (iii) during any subsequent repeat hospitalization.* |

Supplementary table 3: The search strategy of each database.

| **Database** | **Search strategy** | **n, search results** |
| --- | --- | --- |
| **PubMed** | (("Transcatheter Aortic Valve Replacement"[MeSH] OR "Transcatheter Aortic Valve Replacement" OR "TAVR" OR "TAVI")  AND  ("Perclose ProGlide" OR "suture-based closure device" OR "suture-based")  AND  ("Angio-Seal" OR "Angioseal" OR "plug-based closure device" OR "plug-based")) | 33 articles |
| **Cochrane (CENTRAL)** | ("Transcatheter Aortic Valve Replacement" OR "TAVR" OR "TAVI")  AND  ("Perclose ProGlide" OR "suture-based" OR "suture-based closure device")  AND  ("Angio-Seal" OR "Angioseal" OR "plug-based" OR "plug-based closure device") | 9 articles |
| **Scopus** | (TITLE-ABS-KEY("Transcatheter Aortic Valve Replacement" OR "TAVR" OR "TAVI"))  AND  (TITLE-ABS-KEY("Perclose ProGlide" OR "suture-based" OR "suture-based closure device"))  AND  (TITLE-ABS-KEY("Angio-Seal" OR "Angioseal" OR "plug-based" OR "plug-based closure device")) | 52 articles |
| **Web of Science** | ("Transcatheter Aortic Valve Replacement" OR "TAVR" OR "TAVI")  AND  ("Perclose ProGlide" OR "suture-based" OR "suture-based closure device")  AND  ("Angio-Seal" OR "Angioseal" OR "plug-based" OR "plug-based closure device") | 26 articles |

Supplementary table 4. Characteristics of the included studies.

| Author, Year | Country | Study type | Intervention | Comparator | patients, n (intervention/comparator) | Age | male | BMI | Hypertension | DM | CAD | CKD | PVD | AF | Hyperlipidaemia | Stroke or TIA | Hemoglobin | LVEF | Prior MI | prior CABG | prior PCI |
| --- | --- | --- | --- | --- | --- | --- | --- | --- | --- | --- | --- | --- | --- | --- | --- | --- | --- | --- | --- | --- | --- |
| Yeh 2024 | Taiwan | RCT | Single PP + AS | Dual PP | 132/122 | 81.2/82.7 | 60/56 | 24.2/24.2 | 93/80 | 50/43 | 48/41 | 62/53 | 7/8. | 25/21 | 51/49 | 14/6 | 11.6/11.7 | 63.2/64.1 | 0 | NA | NA |
| Rheude 2024 | Germany | RCT | Single PP/ProStyle + AS | Dual PP/ProStyles | 230/224 | 80.1/80.3 | 111/130 | 26.2/27.1 | 186/179 | 68/63 | 164/174 | NA | 26/16 | 89/84 | NA | 20/21 | 12.9+1.8/13.1+1.8 | | 17/23 | 15/16 | 63/76 |
| Sarathy 2021 | UK | Observational | Single PP + AS | Dual PP | 40/46 | 84/86 | NA | 26.1/26.2 | 34/32 | 9/7. | NA | NA | NA | NA | NA | NA | NA | NA | 3/5. | NA | NA |
| Ko 2019 | Taiwan | Observational | Single PP + AS | Dual PP | 100/51 | 80.6/81.8 | 39/28 | 24.3/23 | 65/31 | 43/17 | 37/24 | 31/15 | 14/9 | 17/9 | 30/15 | NA | 11.9/11.7 | 65.1/67.4 | NA | NA | NA |
| Cakal 2022 | Turkey | Observational | Dual PP + AS | Dual PP | 46/139 | 79.07/78.91 | 25/77 | 25.2/26.3 | 44/124 | 14/29 | 32/77 | 21/50 | 9/28. | 10/35. | NA | 5/7. | 11.39/11.54 | 50.76/50.97 | 15/35 | 12/22. | 14/36 |
| Costa 2021 | Italy | observational | Dual PP/Single Prostar + AS | Dual PP/Single Prostar | 123/123 | 82/81 | 47/49 | NA | 107/108 | 45/46 | NA | NA | 6/9. | 18/20 | NA | NA | NA | 55/55 | NA | 6/6. | 12/17. |
| Kiramijyan 2016 | USA | Observational | Dual PP + AS | Dual PP | 208/179 | 82.6 /82.9 | 118 /93 | 27.6/27.5 | 185/158 | 76/54 | 130/101 | 67/74 | 53/55 | 85/78 | 160/138 | 17/21 | NA | NA | NA | 75/45 | 73/48 |

Supplementary table 5. NOS scale for observational studies

| **Study ID** | **Selection (maximum 4)** | | | | **Outcomes selected** | **Comparability (maximum 2)** | **Outcome (maximum 3)** | | | **Total score (maximum 9)** | **Overall appraisal (poor, fair, or good)** |
| --- | --- | --- | --- | --- | --- | --- | --- | --- | --- | --- | --- |
|  |  |  |  |  |  |  |  |  |  |  |  |
|  | **1** | **2** | **3** | **4** | **5** | | **6** | **7** | **8** |  |  |
|  | **Representativeness of exposed cohort** | **Selection of non-exposed cohort** | **Ascertainment of exposure** | **Demonstration that outcome of interest was not present at start of study** | **Comparability of cohorts on the basis of the design or analysis** | | **Assessment of outcome** | **Was follow-up long enough for outcomes to occur** | **Adequacy of follow up of cohorts** |  |  |
|  |  |  |  |  |  |  |  |  |  |  |  |
|  | **(0 or ✵)** | **(0 or ✵)** | **(0 or ✵)** | **(0 or ✵)** | **(0, ✵, or ✵✵)** | | **(0 or ✵)** | **(0 or ✵)** | **(0 or ✵)** |  |  |
| **Cakal 2022** | * | * | * | * | Peripheral vascular disease, Age | ** | * | * | * | 9 | Good |
| **Sarathy 2021** | * | * | * | * | Peripheral vascular disease, Age | * | * | * | * | 8 | Good |
| **Ko 2019** | * | * | * | * | Peripheral vascular disease, Age | * | * | * | * | 8 | Good |
| **Kiramijan 2016** | * | * | * | * | Peripheral vascular disease, Age | * | * | * | * | 8 | Good |
| **Costa 2021** | * | * | * | * | Peripheral vascular disease, Age | ** | * | * | * | 9 | Good |


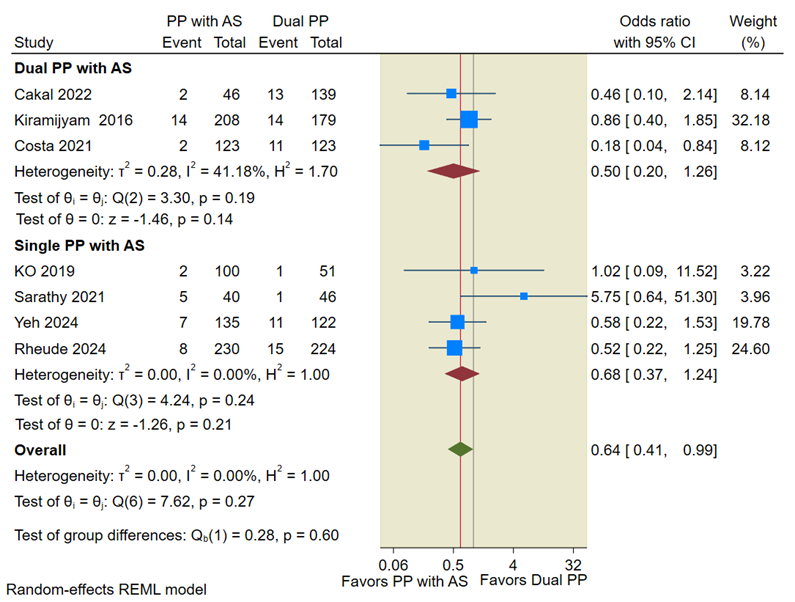


**Supplementary Figure 1:** Forest plot of Major Vascular Complications.


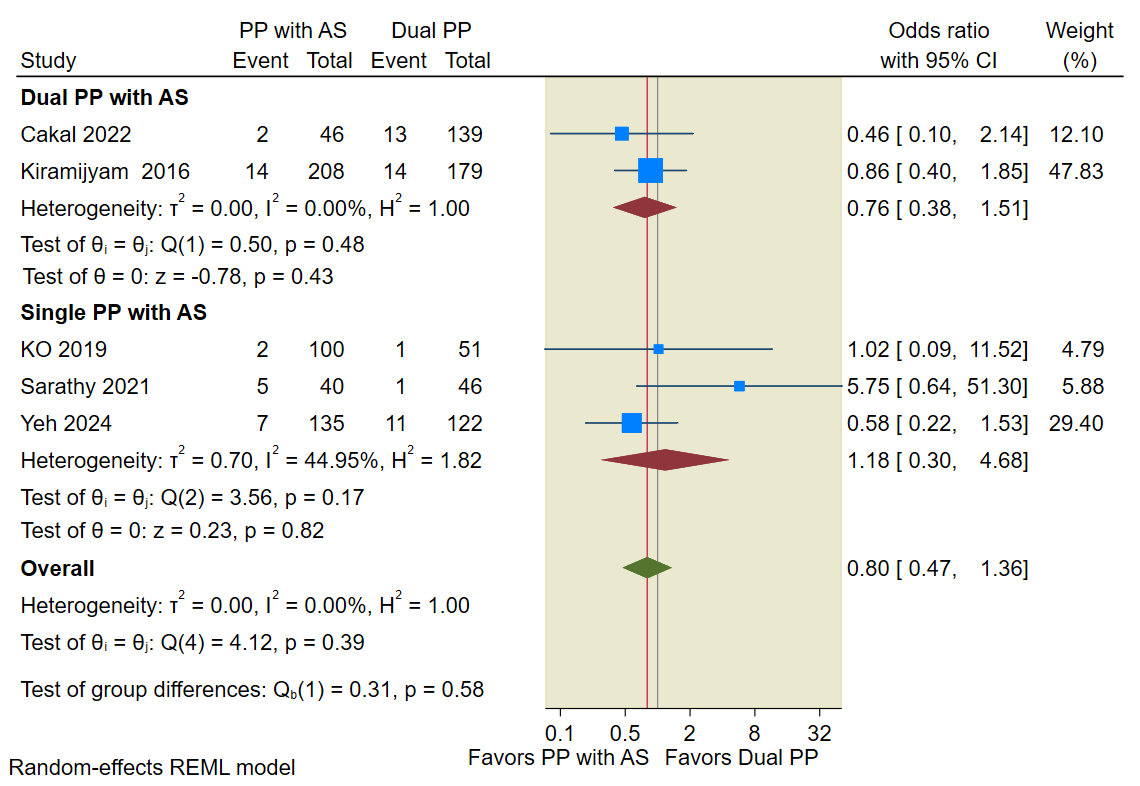


**Supplementary Figure 2:** Sensitivity analysis plot of Major Vascular Complications excluding the studies by Rheude et al. in “single PP with AS” and Costa et al. in “Dual PP with AS”


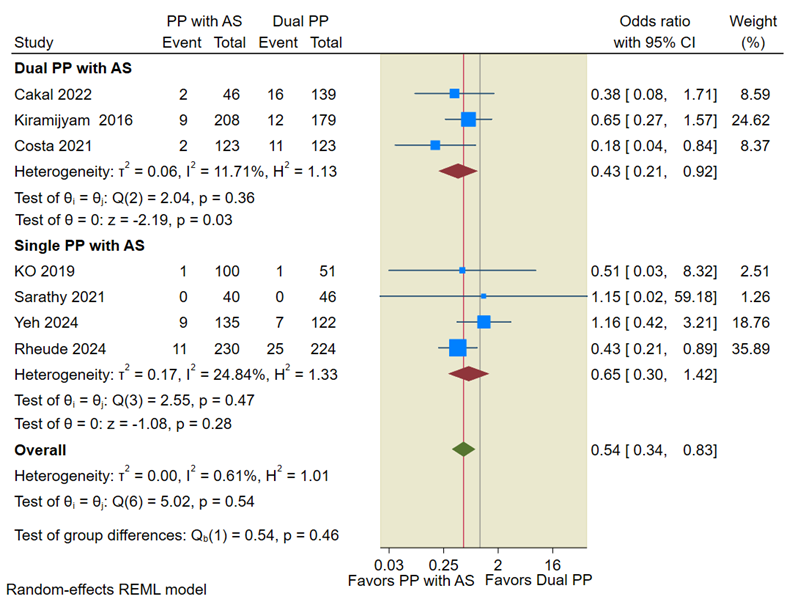


**Supplementary Figure 3:** Forest plot of major bleeding.


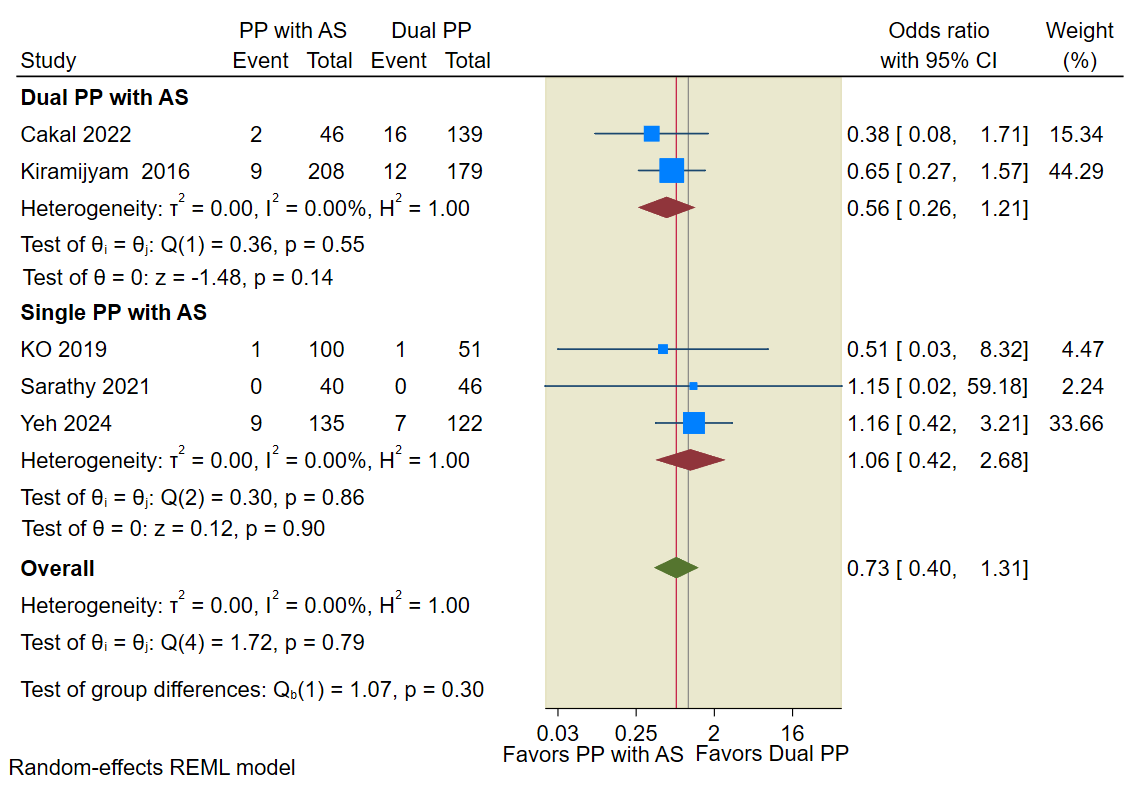


**Supplementary Figure 4:** Sensitivity analysis plot of Major Bleeding excluding the studies Rheude et al. in “single PP with AS” and Costa et al. in “Dual PP with AS”


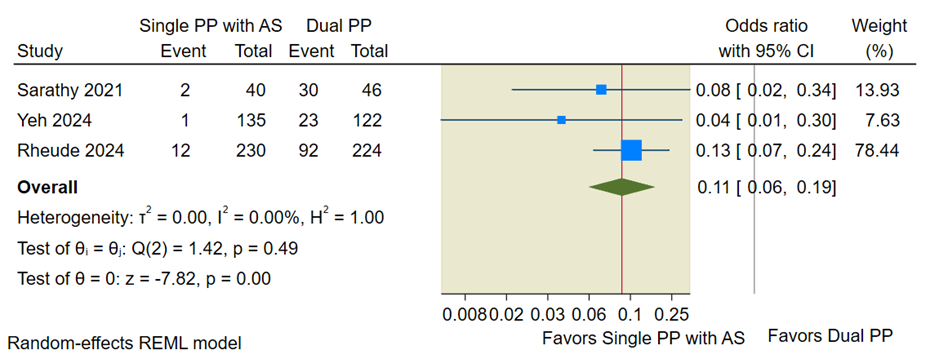


**Supplementary Figure 5:** Forest plot of use of additional VCD.


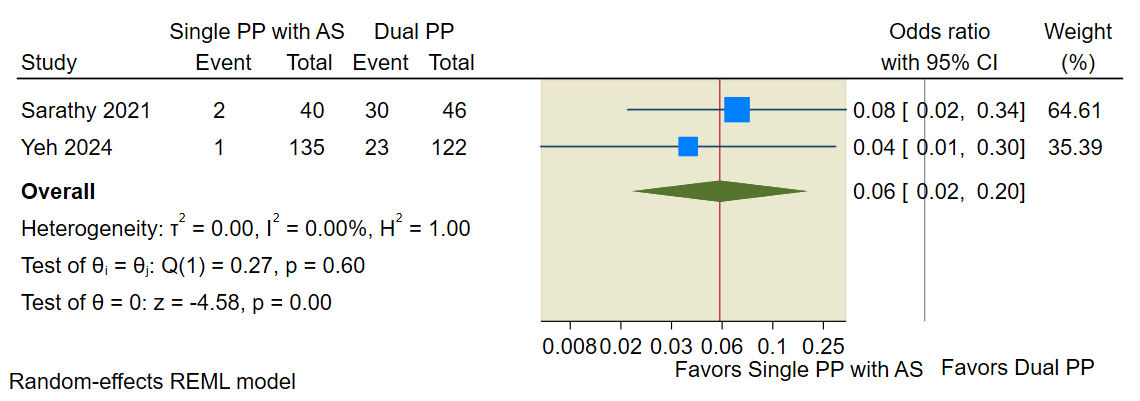


**Supplementary Figure 6:** Sensitivity analysis plot of use of additional VCD excluding the study by Rheude et al.


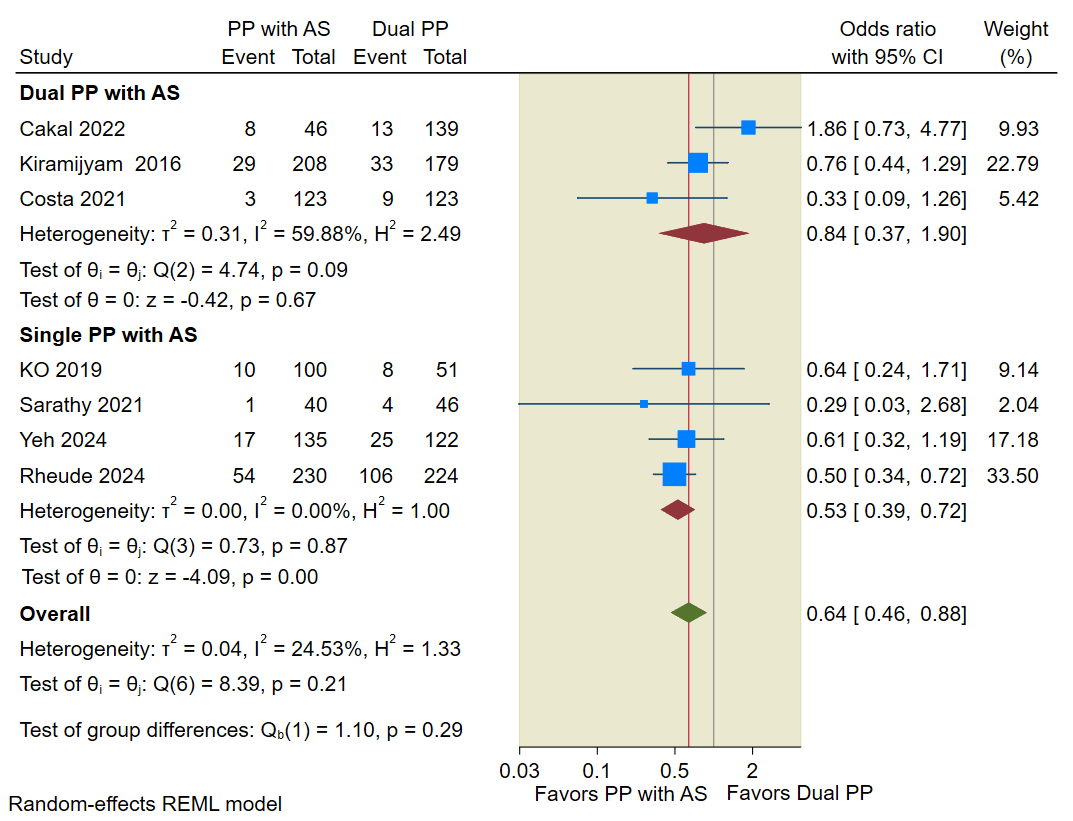


**Supplementary Figure 7:** Forest plot of minor vascular complications


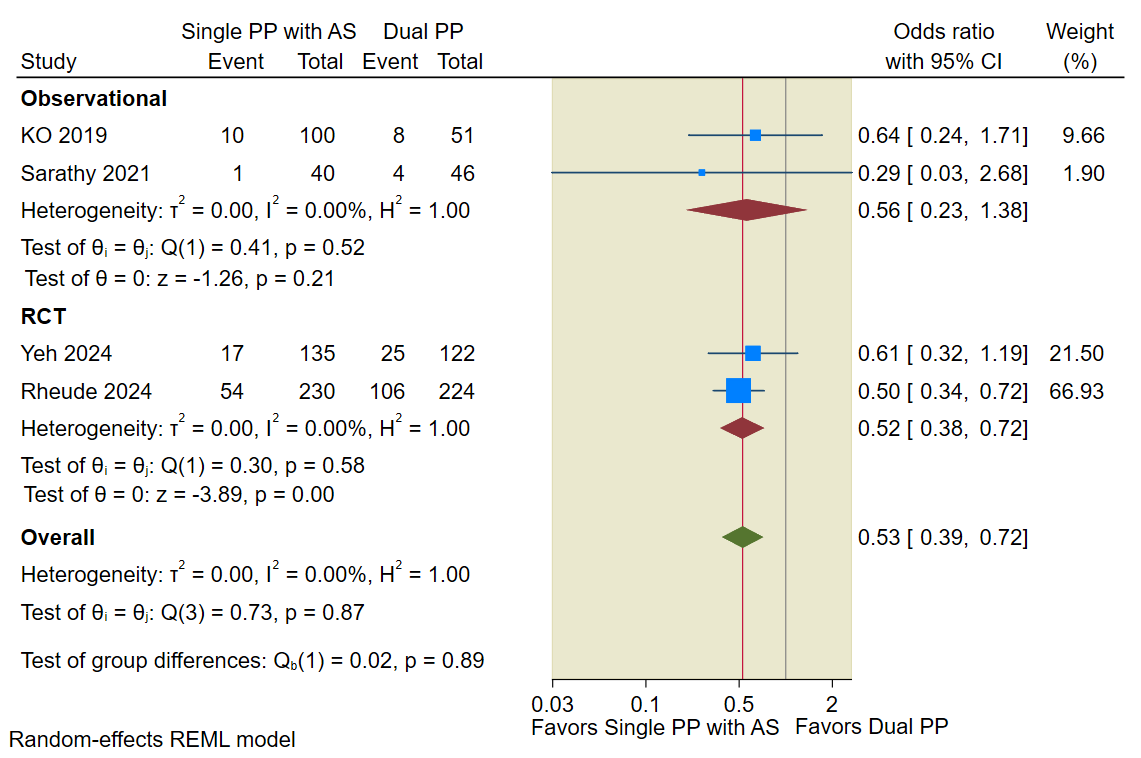


**Supplementary Figure 8:** Forest plot of minor vascular complications according to study design.


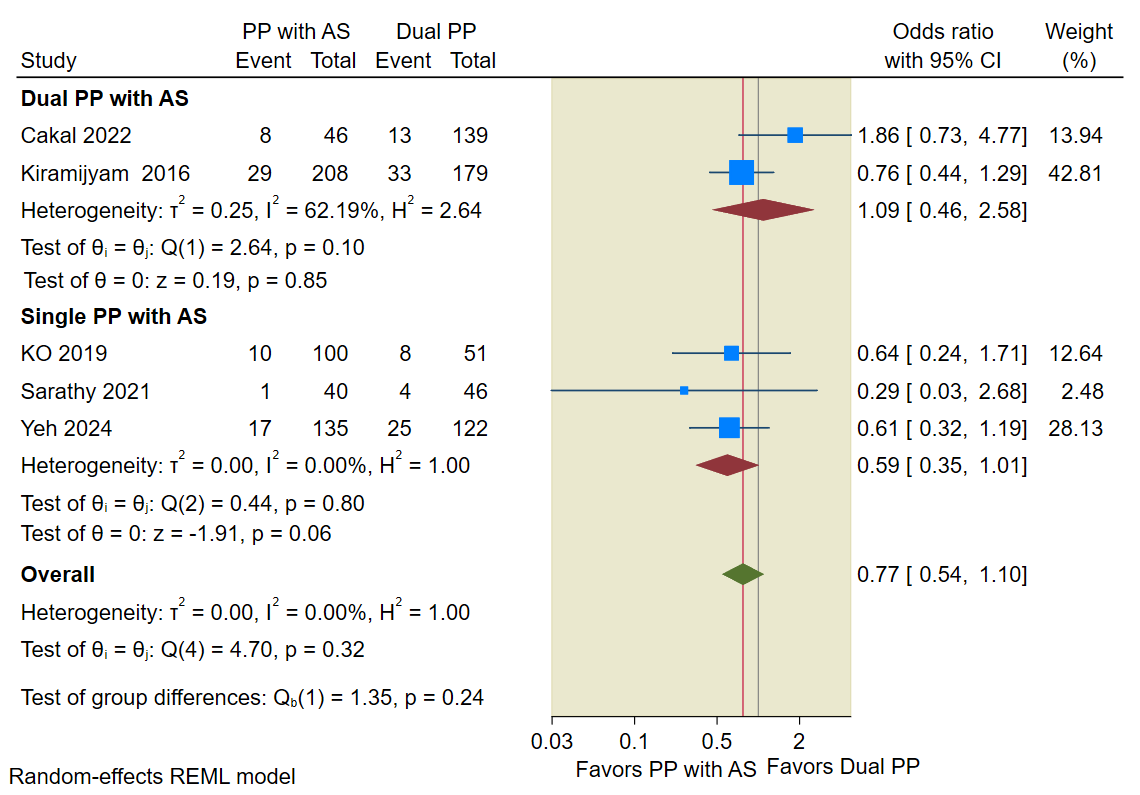


**Supplementary Figure 9:** Sensitivity analysis plot of minor vascular complications excluding the study by Rheude et al.


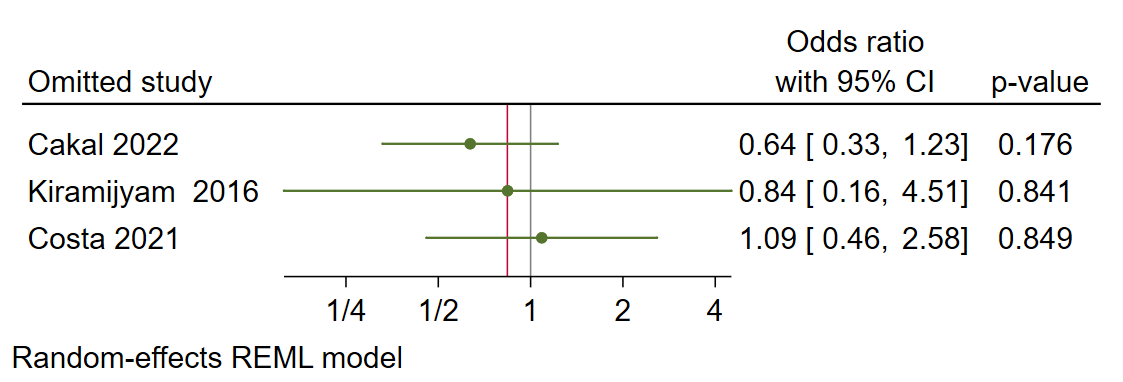


**Supplementary Figure 10:** Leave-one-out plot of minor vascular complications


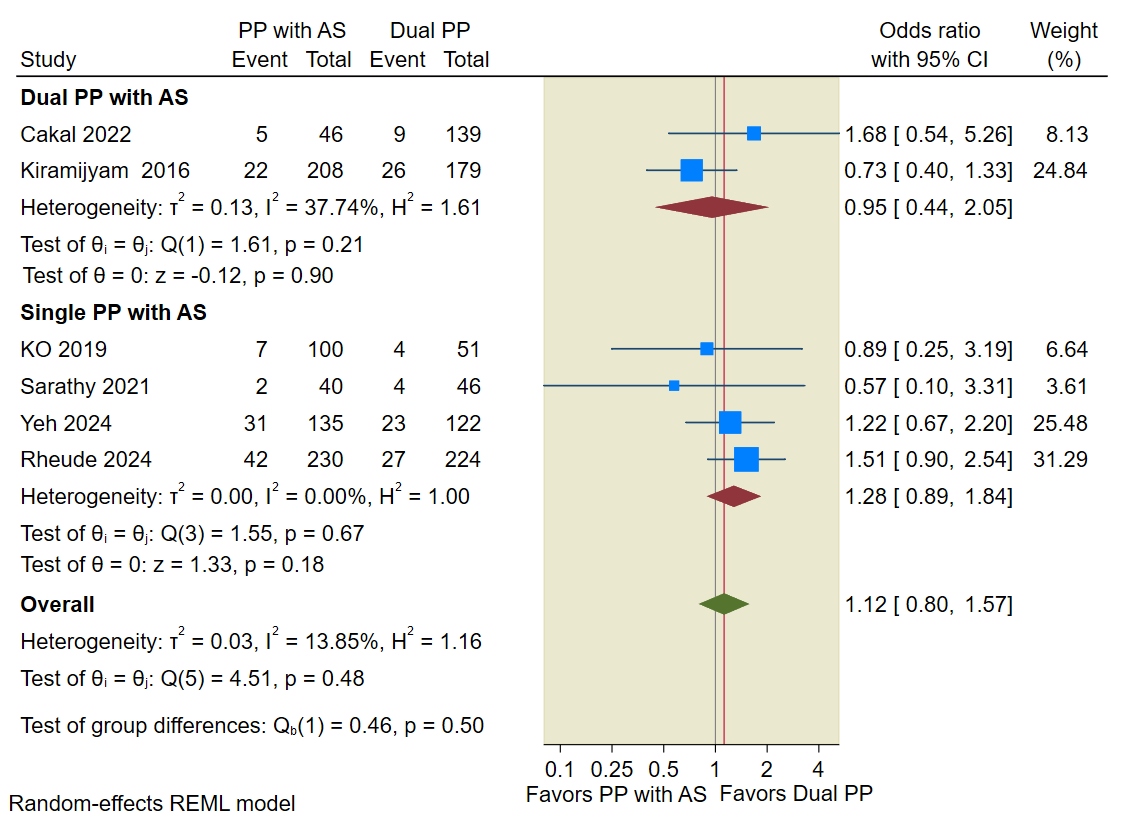


**Supplementary Figure 11:** Forest plot of minor bleeding


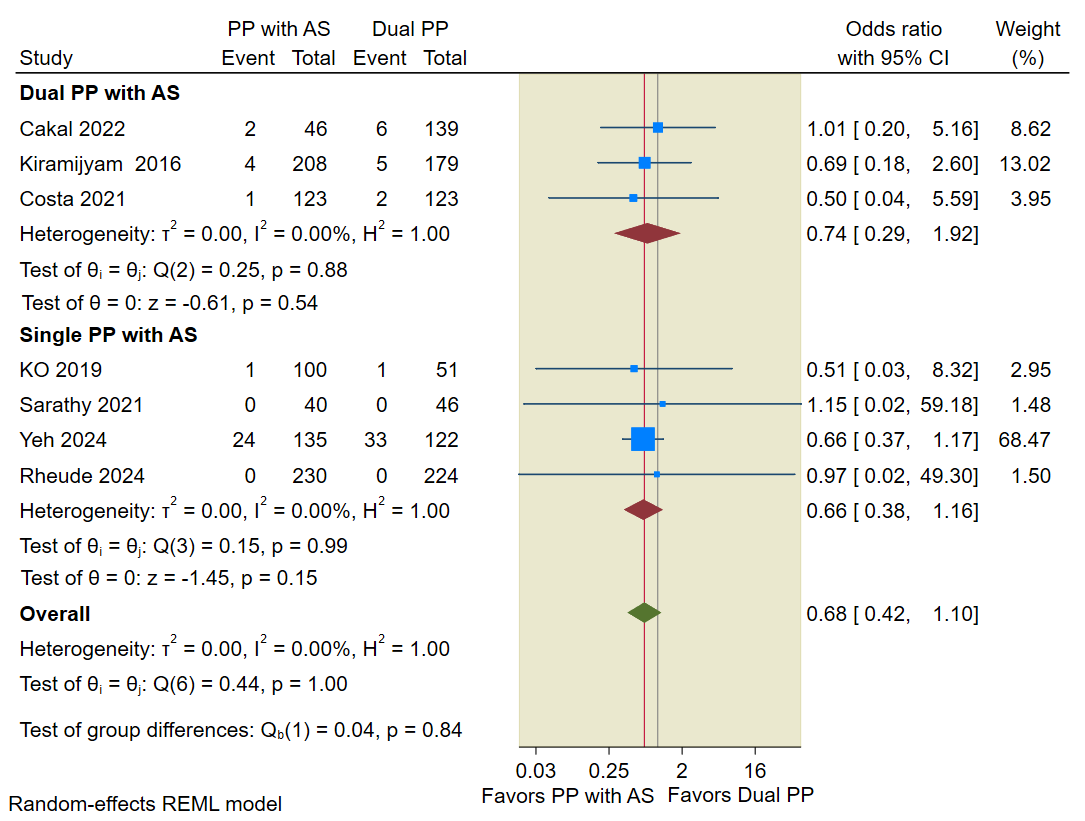


**Supplementary Figure 12:** Forest plot of unplanned surgical intervention


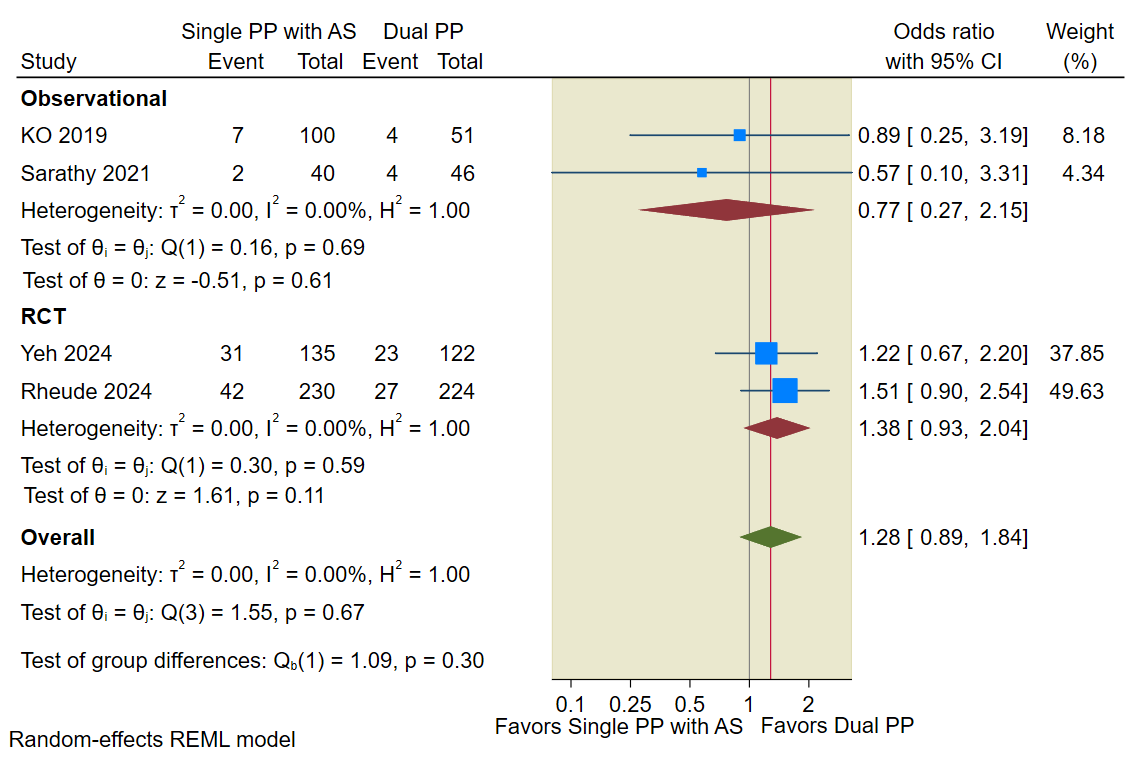


**Supplementary Figure 13:** Forest plot of minor bleeding according to study design.


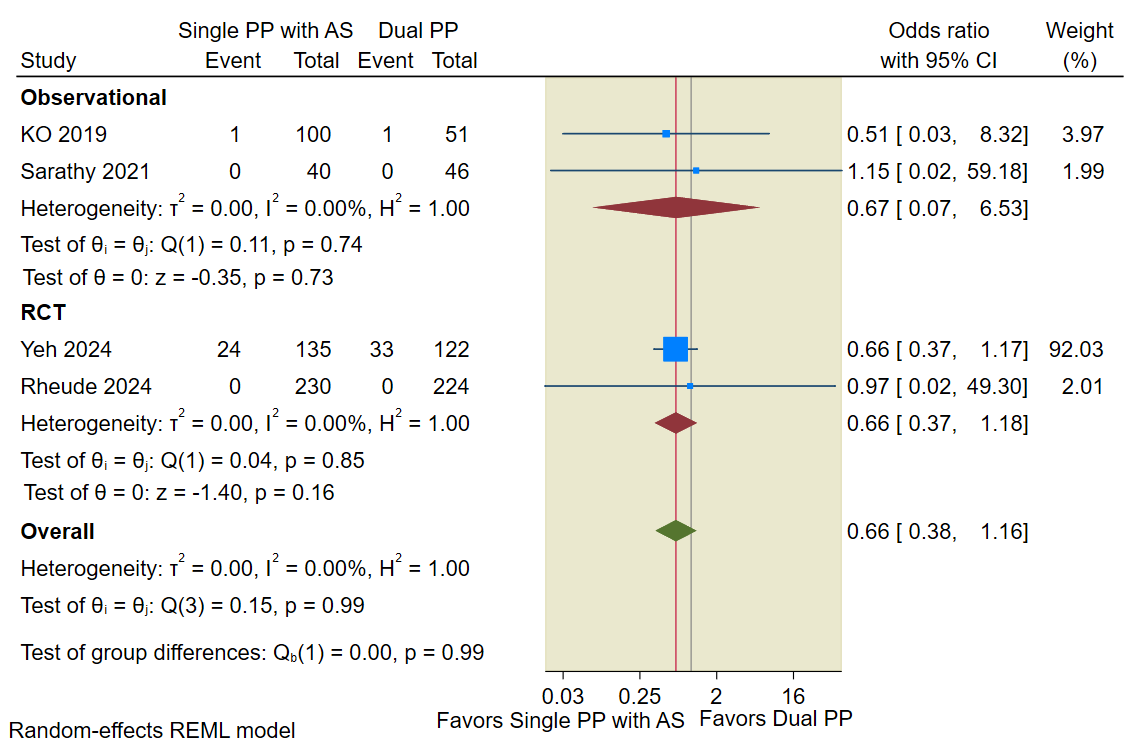


**Supplementary Figure 14:** Forest plot of unplanned surgical intervention according to study design.


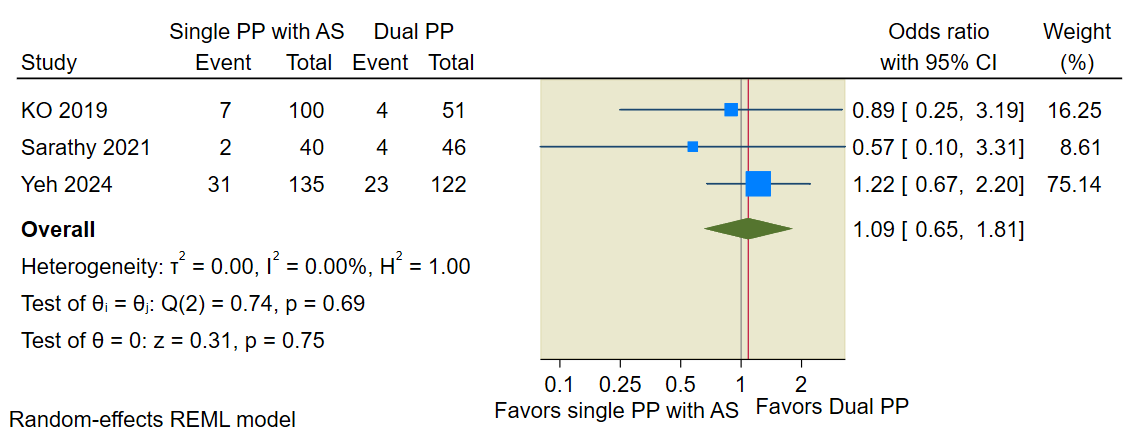


**Supplementary Figure 15:** Sensitivity analysis plot of minor bleeding excluding the study by Rheude et al.


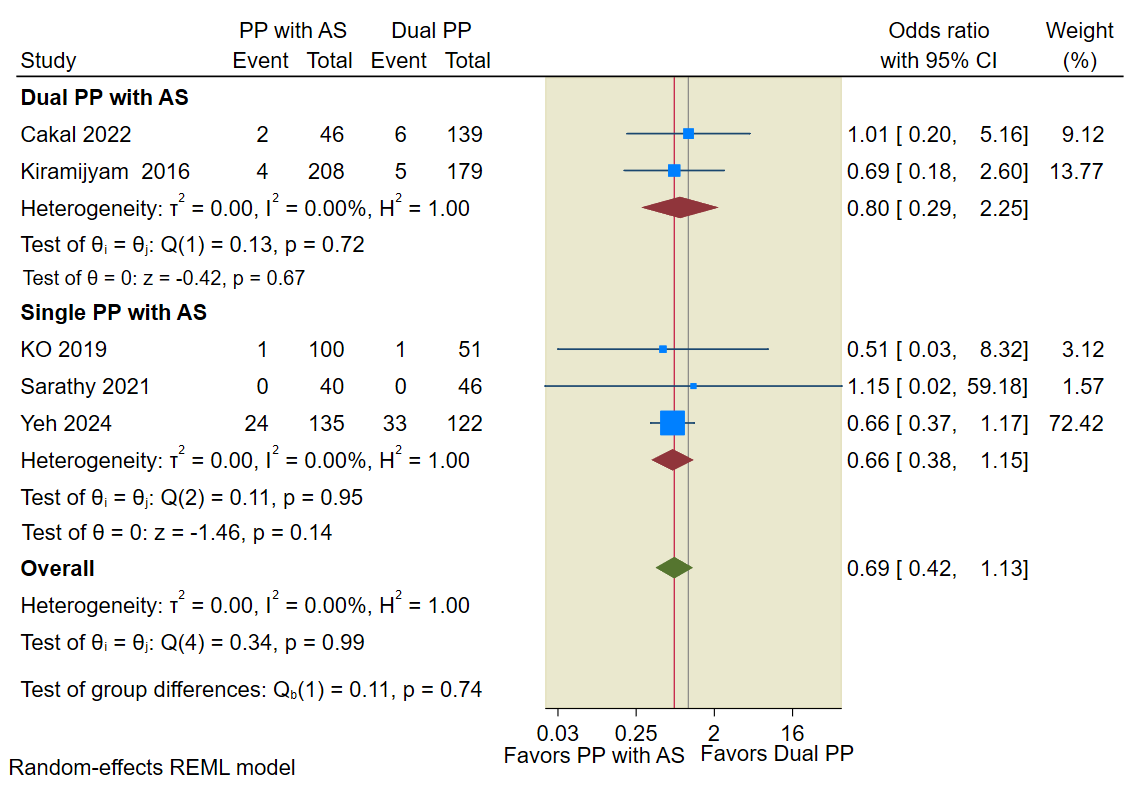


**Supplementary Figure 16:** Sensitivity analysis plot of unplanned surgical intervention excluding the studies Rheude et al. in “single PP with AS” and Costa et al. in “Dual PP with AS”


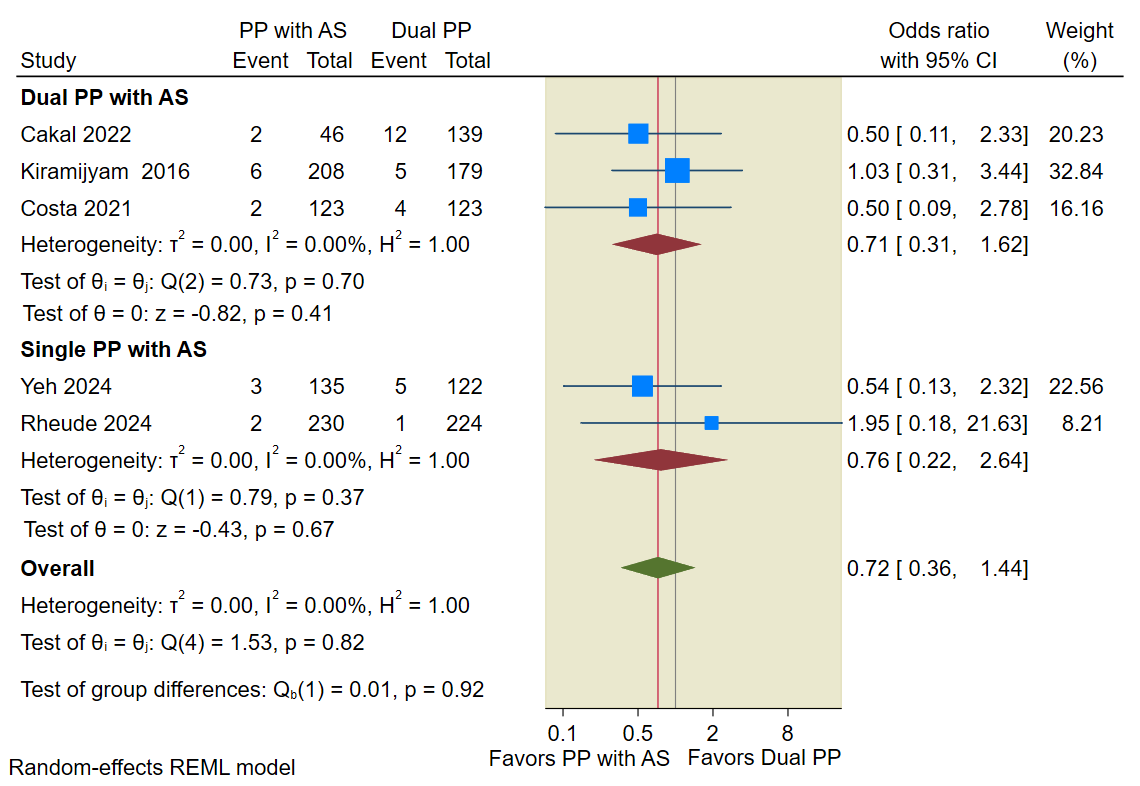


**Supplementary Figure 17:** Forest plot of mortality


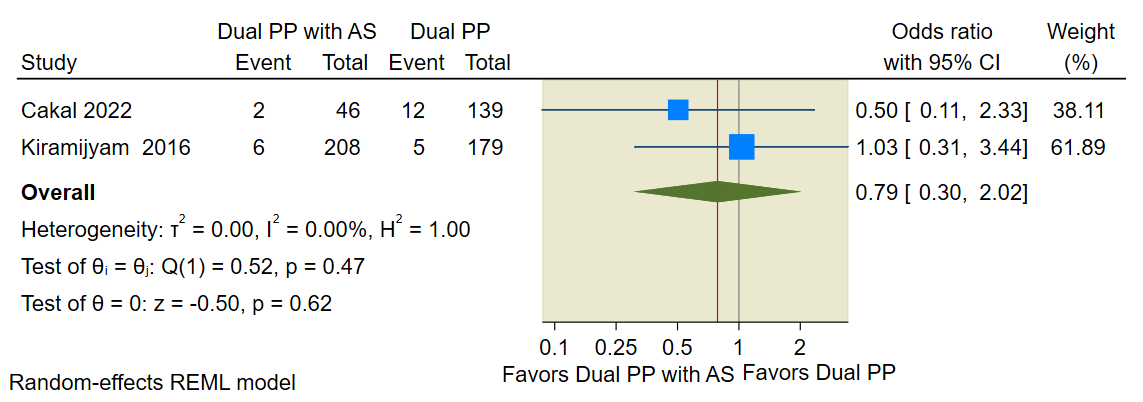


**Supplementary Figure 18:** Sensitivity analysis plot of mortality excluding the study by Costa et al.


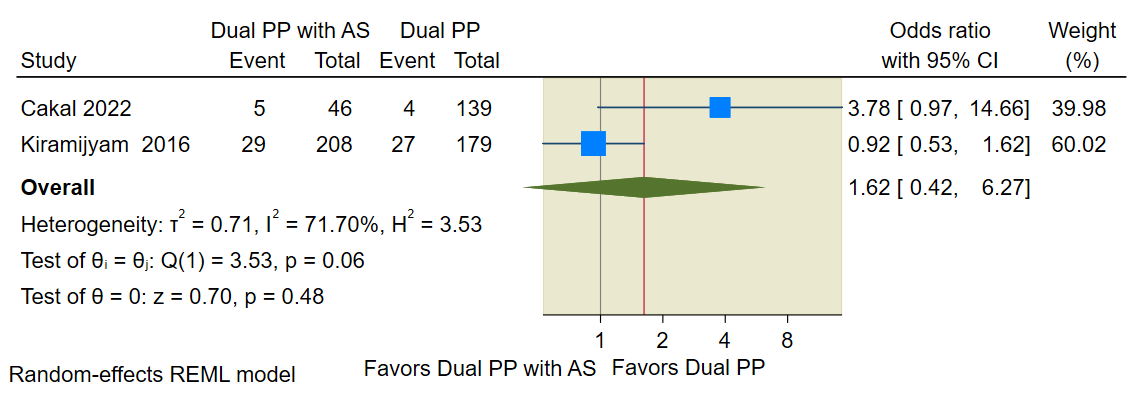


**Supplementary Figure 19:** Forest plot of hematoma


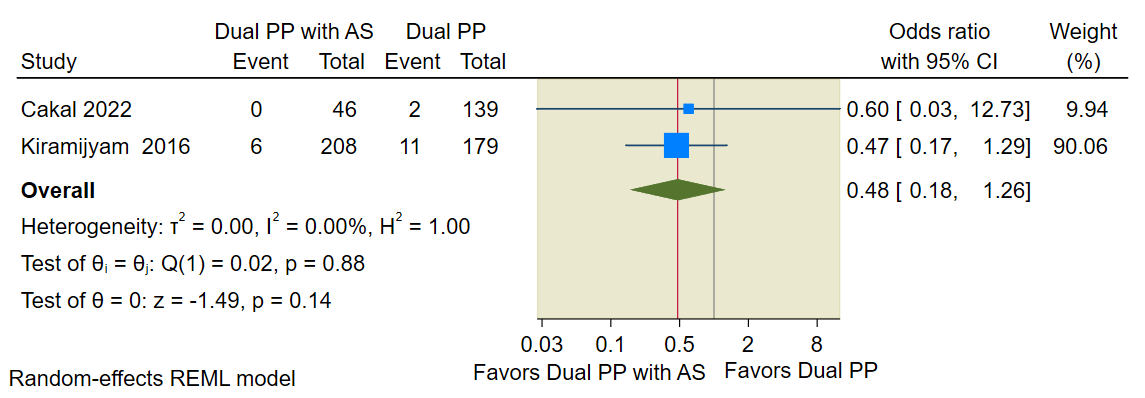


**Supplementary Figure 20:** Forest plot of pseudoaneurysm


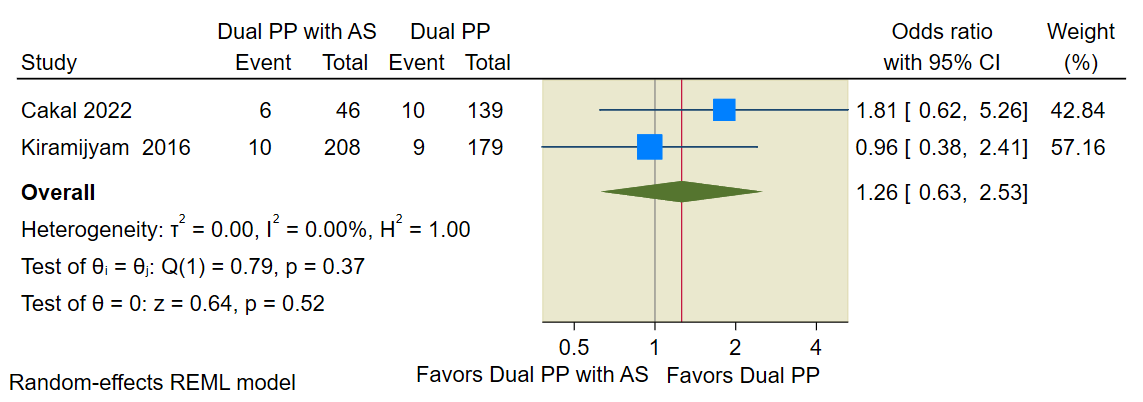


**Supplementary Figure 21:** Forest plot of dissection
